# Contributing Factors to Severe Workplace Violence in Forensic Psychiatry: A Longitudinal Retrospective Analysis of Incident Reports

**DOI:** 10.1111/jpm.70173

**Published:** 2026-07-09

**Authors:** Matias Karvonen, Riitta Askola, Emilia Laukkanen, Anssi Kuosmanen, Lauri Kuosmanen

**Affiliations:** ^1^ Department of Nursing Science University of Eastern Finland Kuopio Finland; ^2^ Health and Social Care Education Unit South‐Eastern Finland University of Applied Sciences Mikkeli Finland; ^3^ Department of Nursing Science University of Turku Turku Finland; ^4^ Savonia University of Applied Sciences Kuopio Finland; ^5^ Niuvaniemi Hospital Kuopio Finland

**Keywords:** forensic psychiatry, incident reporting system, psychiatric hospital, psychiatric nursing, workplace violence

## Abstract

**Background:**

Psychiatric nurses in forensic psychiatric units encounter high levels of workplace violence, but less is known about how ward routines and environmental factors relate to incident severity.

**Aim/Question:**

To identify temporal, routine‐related, work‐related and environmental factors associated with severe reported workplace violence incidents in a Finnish forensic psychiatric hospital.

**Method:**

This longitudinal retrospective study analysed 956 workplace violence reports from 2020–2024 using descriptive statistics, chi‐square tests with Benjamini–Hochberg FDR correction and inductive content analysis of narratives from incidents involving outdoor activities.

**Results:**

Severe reported incidents were associated with time of day (FDR‐adjusted *p* = 0.030, V = 0.12), incidents involving outdoor activities (FDR‐adjusted *p* = 0.030, V = 0.08), incident location (FDR‐adjusted *p* < 0.001, V = 0.28) and ward (FDR‐adjusted *p* < 0.001, V = 0.46). Incidents involving outdoor activities were more often classified as severe and frequently involved physical violence.

**Discussion:**

Daytime and afternoon periods, incidents involving outdoor activities and specific ward locations were associated with a higher proportion of severe reported incidents, suggesting that routine and environmental factors shape incident severity.

**Limitations:**

Limitations include the single‐site retrospective design, voluntary reporting, possible repeated incidents involving the same patients and subjective classification of some reports.

**Implications for Practice:**

Targeted nurse‐led prevention should focus on incidents involving outdoor activities, afternoon peaks and location‐specific controls to support safety in forensic psychiatric wards.

## Key Points

- Severe workplace violence in forensic psychiatric nursing clustered around specific routines, times, locations and wards.
- Outdoor activities emerged as a distinct high‐risk routine, with incidents more often severe and physically violent.
- Prevention should target dynamic risk screening, staffing and environmental controls during high‐activity periods and in high‐risk ward locations.

## 1 Introduction

Working in forensic psychiatric wards exposes psychiatric nurses to various forms of workplace violence including verbal, physical and sexual violence (Ireland et al. 2021; Newman et al. 2021, 2024). Previous studies indicate that 17%–40% of forensic psychiatric patients engage in violent behaviour during hospitalisation (Broderick et al. 2015; Eisele et al. 2021; Moscovici et al. 2024). According to research, the majority (69%) of workplace violence caused by patients is directed towards other patients (Bader et al. 2014), but a significant share (70%–100%) of forensic nursing staff members have experienced violence in the past year (Kelly et al. 2015; Niu et al. 2019; Newman et al. 2024). A review study found that 31%–42% of forensic psychiatric inpatients displayed aggressive behaviour during hospitalisation (Newman et al. 2021). Notably, most incidents of violence in forensic psychiatric wards are caused by a small group of patients (Broderick et al. 2015). Taken together, these findings indicate that workplace violence in forensic psychiatry is not an exceptional event but a recurrent feature of the care environment, making it important to identify not only who is at risk, but also when, where and under what routine conditions severe incidents occur.

Multiple factors underlie violent behaviour in forensic psychiatric patients. Developmental, clinical and interpersonal risk factors include previous exposure to violence, unstable living conditions, personal or family experiences of violence and abuse, psychiatric diagnoses, substance abuse and personality traits such as lack of empathy and impulsivity (Bader and Evans 2015; Fosse et al. 2021; Wolf et al. 2023). However, patient‐related factors alone do not fully explain why violent incidents cluster around particular times, activities or locations (Bader et al. 2014; Weltens et al. 2021; Kuosmanen et al. 2022). Recent research on staff‐targeted transgressive incidents in forensic psychiatric healthcare also suggests that incident severity and impact vary according to patient, staff and contextual characteristics (Frowijn et al. 2024). Therefore, this study draws on a situational framework informed by routine activity theory, its later developments on guardianship and place management and environmental psychology. From this perspective, severe workplace violence may be more likely when an aroused or distressed patient, a staff member in close proximity and limited immediate supervision, guardianship or containment coincide within specific ward routines and physical spaces (Cohen and Felson 1979; Schaefer 2021; Weltens et al. 2021; Ross et al. 2022). From this perspective, routines such as outdoor time and locations such as patient rooms, seclusion rooms and day rooms can be understood as recurrent risk situations in which patient vulnerability, staff–patient interaction and environmental constraints intersect.

In forensic psychiatric care settings, patient‐perpetrated workplace violence and aggression adversely affect the physical and mental health of forensic psychiatric nurses (van Leeuwen and Harte 2017; Newman et al. 2024; Bloemendaal et al. 2024) and are associated with PTSD symptoms (Ireland et al. 2021; Jankovic et al. 2021), burnout, stress and reduced psychological well‐being (Bader et al. 2014; Ireland et al. 2021; Kobayashi et al. 2020; Bogaerts et al. 2021). Workplace violence experienced by forensic psychiatric nurses is associated with the number of sick‐leave days taken and with poorer physical health and mental well‐being among staff (Newman et al. 2024). Additionally, violence by forensic psychiatric patients may hinder treatment progress and disrupt the ward's therapeutic climate (Verstegen et al. 2017; Aerts et al. 2023).

Managing the risk of violence in forensic psychiatric units requires organisational‐level structures, clear policies and procedures and systematic staff training. Leadership, staff approaches, ward environment and post‐incident debriefing play a key role in violence prevention and management in psychiatric inpatient care (Asikainen et al. 2020). Evidence for de‐escalation training is promising yet somewhat mixed; nevertheless, it remains a core component of overall safety (Brenig et al. 2023; Johnston et al. 2022). Structured violence‐risk assessment (e.g., DASA, HCR‐20) and systematic incident recording improve anticipation, learning and decision‐making (Ogonah et al. 2023). By training healthcare staff to handle violent situations, threats and risks, these situations can be effectively prevented (Cowman et al. 2017).

Reporting workplace violence incidents is important in order to identify violence risk factors and to respond to them. In Finland, electronic incident reporting systems are widely used in social and healthcare services to record patient and occupational safety incidents, including workplace violence, near misses and adverse events (Hyvämäki et al. 2023). This study used workplace violence incident reports to examine when, where and during which activities severe incidents occurred.

Previous research has shown that daily routines and events on psychiatric wards may be more strongly associated with patient aggression than simply the time of day or day of the week (Bader et al. 2014; Kuosmanen et al. 2022). In this study, forensic psychiatric nurses' work tasks refer to role‐bound activities at patient and unit levels (e.g., patient care, medication management, documentation) (Abt et al. 2022; Glanz et al. 2023). Daily ward routines in forensic psychiatry are understood as time‐bound or recurrent arrangements (e.g., outdoor time, mealtimes) that structure the functioning of the care environment (QNFMHS 2021). Together, previous research and the situational framework outlined above suggest that the severity of reported workplace violence may be shaped by active ward periods, recurrent routines and environmental constraints. However, previous findings on temporal patterns are not fully consistent and time of day may partly reflect exposure to ward activities rather than an independent risk factor. Therefore, further research is needed to clarify how routines, activities and environmental conditions are associated with the severity of workplace violence against psychiatric nurses in forensic inpatient care.

## 2 Aims

The aim of this study was to identify temporal, routine‐related, work‐related and environmental factors associated with the severity of reported workplace violence incidents affecting psychiatric nursing staff in a Finnish forensic psychiatric hospital.

The following research questions were addressed:

1. What is the relationship between daily routines and everyday ward activities and the severity of reported workplace violence incidents in forensic psychiatric inpatient care?
2. What work and environmental factors trigger workplace violence in forensic inpatient care?

Guided by the situational framework outlined above and previous research, four hypotheses were formulated to examine whether severe reported workplace violence incidents were associated with temporal, routine‐related and environmental factors (Table 1).

**TABLE 1:** Study hypotheses.

Hypothese 1	The proportion of reported incidents classified as severe is higher during active daytime and afternoon periods than during evening or night‐time hours (Kuosmanen et al. 2022).
Hypothese 2	Incidents involving outdoor activities are more likely to be classified as severe than incidents not involving outdoor activities (Kuivalainen et al. 2014; Bader et al. 2014).
Hypothese 3	Incidents occurring in patient rooms, seclusion rooms, outdoor areas and day rooms are more likely to be classified as severe than incidents occurring in administrative or staff‐only areas (Weltens et al. 2021; Kuosmanen et al. 2022).
Hypothese 4	The proportion of reported incidents classified as severe differs across wards, reflecting differences in patient case mix, security level and routine care practices (Frowijn et al. 2024).

## 3 Materials and Methods

### 3.1 Design and Settings

This study was conducted as a longitudinal retrospective analysis of workplace violence incident reports retrieved from the HaiPro patient and occupational safety reporting system used by psychiatric nurses at a Finnish forensic psychiatric hospital. The hospital provides specialised forensic psychiatric services and conducts forensic psychiatric evaluations nationwide (Kuosmanen et al. 2013).

In Finland, a person is classified as a forensic psychiatric patient if their involuntary treatment has been initiated by order of the Finnish Institute for Health and Welfare (THL) in connection with criminal proceedings, following a forensic psychiatric assessment or a treatment needs evaluation. There are approximately 400 such patients in Finland. Of those receiving treatment in forensic psychiatric hospitals, about 60% are patients ordered into treatment by THL, while the remaining 40% are patients admitted under the Mental Health Act (Mental Health Act (1116/1990) (Finland) 1990) due to psychiatric disorders that require particularly intensive or high‐risk care (Seppänen et al. 2020).

### 3.2 Data Collection

The data were collected from web‐based workplace violence reports logged in the HaiPro database. All eligible incident reports recorded at the study hospital between 1 January 2020 and 31 December 2024 were consecutively included as an event‐based census; duplicates and records with critical missing fields were excluded using prespecified rules. This complete 5‐year inclusion provides a comprehensive description of reported WPV incidents across seasons, shifts and routine activities at the study hospital. In total, 1018 reports were identified and 956 were analysed.

A workplace violence incident is reported in the HaiPro system as an occupational safety report. Staff from all professional groups can submit occupational safety reports. In the report, the incident is classified as either a ‘near miss’, a situation that could have led to an undesired event or as an ‘incident/injury to staff’, where a violent act was directed at staff, such as being hit or pushed by a patient. In an occupational safety report, the person making the report specifies the unit where the violent incident occurred, the time and location of the event, their professional group and the type of hazard. In all the occupational safety reports used in this study, the reported hazard type is ‘violence’.

The person reporting the incident/observation writes a free‐text description of what happened, including what task they were performing when the violent situation arose and a narrative of how the event unfolded. Next, the reporter describes the individuals involved in the workplace violence incident and who was called to the scene as a result. Finally, the reporter gives a verbal account of the factors that may have contributed to the workplace violence incident. A HaiPro report is not made solely for the purpose of recording threats and hazardous situations. The primary goal is for the work community to process the incidents and learn from them (Awanic 2020).

### 3.3 Ethical Considerations

The reports do not contain patient or staff names or indirect identifiers to protect privacy and the research material has been processed in statistical form. A privacy statement and data protection impact assessment have been prepared for the study. Based on Finnish legislation, ethical approval was not needed (Finnish National Board on Research Integrity TENK 2019). The data for the study were obtained by applying for a research permit from the hospital in question. The permit was granted by the hospital's chief physician and the data were provided to the researcher by the hospital's security manager. The hospital under study has granted the researcher permission to conduct the research.

### 3.4 Variable Operationalisation and Coding Reliability

Study variables were operationalised using structured HaiPro fields and free‐text narratives. Time of day, day of week, ward, incident location, reporter's professional group and incident outcome were derived from structured fields. Workplace violence severity was operationalised as a binary variable based on the reported type of violence and the classification by Kelly et al. (2015): category 1: Mild incidents of workplace violence (e.g., verbal abuse, shouting, destruction of property, sexual harassment) and category 2: Severe incidents of workplace violence (e.g., biting, hitting, kicking, swinging, threats involving a knife, strangulation).

Reports initially classified as ‘Other situation, specify?’ (*n* = 68) were manually reviewed and recoded by one researcher as either mild or severe workplace violence based on the free‐text description; the original HaiPro event‐type category was retained for descriptive reporting. Recoding was guided by the predefined severity criteria above and the same decision rules were applied throughout the dataset. Because formal interrater reliability could not be assessed, some risk of classification bias remains.

### 3.5 Data Analysis

During the 5‐year period, hospital staff submitted 1018 workplace violence reports in the HaiPro database, 956 of which were included in this analysis (31 were excluded because they did not address workplace violence by patients, 12 because they were duplicates and 19 because the reports were submitted by non‐nursing staff).

Quantitative data were analysed using IBM SPSS Statistics version 29.0.2. Descriptive statistics were used to summarise incident characteristics. Associations between categorical variables were examined using chi‐square tests, with statistical significance set at *p* < 0.05. Expected cell frequencies were inspected before interpreting chi‐square tests and analyses involving sparse cells were interpreted cautiously. To account for multiple testing across the chi‐square tests reported in the quantitative analyses, *p*‐values were adjusted using the Benjamini–Hochberg false discovery rate (FDR) procedure (Benjamini and Hochberg 1995). This approach was chosen because it controls the expected proportion of false discoveries while being less conservative than the Bonferroni correction, thereby reducing the risk of overlooking potentially relevant associations in hypothesis‐driven analyses. Both unadjusted and FDR‐adjusted *p*‐values are reported, with statistical significance evaluated using FDR‐adjusted *p* < 0.05. Effect sizes were reported using Cramér's *V* where applicable. Because several chi‐square tests were conducted, findings with small effect sizes were interpreted cautiously in relation to the theoretical framework and previous research.

Because this retrospective event‐based census included all eligible reports, the sample size was fixed by the available data. Therefore, no prospective sample size calculation was used to determine recruitment. To assess the adequacy of the available sample for the main hypothesis‐driven chi‐square analyses, a supplementary G*Power assessment was conducted using chi‐square tests for contingency tables, observed Cramér's *V* values, 80% power and *α* = 0.05. The minimum sample sizes required for 80% power were *N* = 829 for time of day, *N* = 1227 for incidents involving outdoor activities, *N* = 240 for incident location and *N* = 85 for ward. Thus, the available sample met the estimated requirement for time of day, incident location and ward, but not for the very small association between incidents involving outdoor activities and severity.

In addition, open‐ended HaiPro narratives were analysed using inductive content analysis with ATLAS.ti. All reports of incidents involving outdoor activities identified in the 5‐year dataset were included; thus, the qualitative sample size was determined by the event‐based census rather than purposive sampling. Incidents involving outdoor activities were defined as reports in which the narrative linked the incident to outdoor activities before, during or immediately after outdoor time, including conflicts originating outdoors but escalating after return to the ward. From this predefined subgroup (*n* = 72), relevant expressions were extracted, condensed and coded, resulting in 87 condensed meaning units. The analysis involved open coding, categorisation and abstraction to identify triggers contributing to severe workplace violence during patients' outdoor activities. Because the aim was to describe and quantify triggers within this predefined subgroup, data saturation was not used as a stopping criterion. Coding was conducted by one researcher using predefined analytic steps and formal interrater reliability was not assessed, which is acknowledged as a methodological limitation. The qualitative findings were also quantified descriptively by summarising the timing, trigger factors, form of violence and outcome of incidents involving outdoor activities (Elo et al. 2014).

## 4 Results

### 4.1 Descriptive Overview of the Data

#### 4.1.1 Annual Distribution and Types of Workplace Violence

A total of 1018 workplace violence incidents were reported in the hospital over a 5‐year period, of which 956 were included in this study. Annual incident counts ranged from 159 to 234, corresponding to 1.6–2.3 incidents per 1000 patient days (based on billed/net patient days). The annual number of workplace violence incidents and the forms of violence are presented in Table 2.

**TABLE 2:** Types of violence and distribution by year.

Types of violence	Total reports	2020	2021	2022	2023	2024	%
Insulting/name‐calling/verbal threats/yelling	235	38	41	60	57	39	24.6%
Pushing/shoving/waving away	124	26	21	31	24	22	13.0%
Breaking/throwing/slamming objects, items or equipment	109	18	31	17	21	22	11.4%
Scratching/biting/spitting/bruising/hitting/kicking	343	57	48	98	74	66	35.9%
Sexual harassment/inappropriate touching	46	0	20	7	8	11	4.8%
Knocking over/preventing movement/holding down/choking	24	6	8	3	4	3	2.5%
Threatening with a weapon/syringe/knife	5	2	0	0	1	2	0.5%
Theft or attempted robbery of money or items	2	0	1	0	0	1	0.2%
Other situation (e.g., threatening demeanour, primarily self‐harming behaviour)	68	9	16	13	14	16	7.1%
Total Reports	956	156	186	229	203	182	100%

#### 4.1.2 Professional Background of Reporters and Characteristics of Incidents

Of the 956 analysed WPV reports, most (78.3%) were reported by registered nurses, followed by practical (mental) nurses (21.7%). Other professional groups (*n* = 19 reports), such as doctors or psychologists, were excluded from the study data. A chi‐square test found no significant association between the reporter's professional group and workplace violence severity, *χ* ^2^(1, *N* = 956) = 0.53, *p* = 0.468, FDR‐adjusted *p* = 0.468, Cramér's *V* = 0.02.

#### 4.1.3 Nature and Consequences of Events (Near Misses vs. Actual Harm)

Of all reported workplace violence incidents, 56.1% involved harm to staff (i.e., actual violent events) and 43.9% were near misses. Severity was significantly associated with the nature of the incident (near miss vs. harm), *χ* ^2^(1, *N* = 956) = 70.45, *p* < 0.001, FDR‐adjusted *p* < 0.001, Cramér's *V* = 0.27, indicating a small‐to‐moderate effect size: 58% of mild incidents were near misses, whereas only 31% of severe incidents were near misses (therefore, 69% of severe incidents resulted in harm to staff). Analyses of harm levels were restricted to reports with a completed ‘consequence to staff’ field (*N* = 535/956; 55.9%); the remaining 421 reports (44.1%) lacked this field and were excluded from harm‐level analyses. In this subset, the association between severity (mild vs. severe) and harm level (none, mild, moderate) was not statistically significant, *χ* ^2^(2, *N* = 535) = 4.82, *p* = 0.090, FDR‐adjusted *p* = 0.165, Cramér's *V* = 0.09. However, moderate harm was slightly more frequent in severe incidents (10.2%) than in mild incidents (8.8%).

### 4.2 Relationship Between Daily Routines, Everyday Ward Activities and the Severity of Reported Workplace Violence Incidents in Forensic Psychiatric Inpatient Care

#### 4.2.1 Workplace Violence Across Times of Day and Day of Week

Workplace violence incidents were reported significantly more often during the daytime than at night. Nearly two‐thirds (64%) of the incidents occurred between 11:01 a.m. and 7:00 p.m. Only 3% of the incidents took place during the night, between 11:01 p.m. and 7:00 a.m.

A chi‐square test of independence was conducted to examine the relationship between the time of day (night, morning, daytime, afternoon, evening) and the severity of workplace violence. The result was statistically significant, *χ* ^2^(4, *N* = 889) = 12.74, *p* = 0.013, FDR‐adjusted *p* = 0.030, Cramér's *V* = 0.12, indicating a small effect size. The proportion of severe workplace violence was highest during the afternoon (57.6%) and daytime (54.7%) hours, whereas lower proportions were observed during the night (42.3%) and evening (40.4%). A chi‐square test found no significant association between time of day and severity of consequences to staff (no harm, mild harm, moderate harm), *χ* ^2^(8, *N* = 500) = 8.44, *p* = 0.392, FDR‐adjusted *p* = 0.431, Cramér's *V* = 0.09. No statistical association was found between the day of the week and workplace violence severity, *χ* ^2^(7, *N* = 956) = 10.18, *p* = 0.179, FDR‐adjusted *p* = 0.246, Cramér's *V* = 0.10.

#### 4.2.2 Routine Ward Activities and Incident Severity

In analysing the HaiPro violent incident reports, those related to patients' outdoor time, mealtimes and medication administration were particularly prominent; therefore, we used chi‐square tests to examine the associations between these activities and severe workplace violence incidents. Incidents involving outdoor activities had a higher proportion of severe cases (65.4%) than incidents not involving outdoor activities (50.8%), *χ* ^2^(1, *N* = 956) = 6.11, *p* = 0.013, FDR‐adjusted *p* = 0.030, Cramér's *V* = 0.08, indicating a small effect size. For mealtimes, no statistically significant association with incident severity was found, *χ* ^2^(1, *N* = 956) = 2.45, *p* = 0.117, FDR‐adjusted *p* = 0.185, Cramér's *V* = 0.05. Similarly, medication distribution was not significantly associated with incident severity, *χ* ^2^(1, *N* = 956) = 1.38, *p* = 0.240, FDR‐adjusted *p* = 0.293, Cramér's *V* = 0.04.

### 4.3 Work and Environmental Factors Triggering Severe Workplace Violence in Forensic Psychiatric Inpatient Care (Nursing Staff's Perspective)

#### 4.3.1 Incident Location and Severity

The highest number of workplace violence incidents were reported to have occurred in ward corridors (30%), patient rooms (25%), seclusion rooms (19%) and day rooms (8%) (Table 3). A chi‐square test found a significant association between incident location and workplace violence severity, *χ* ^2^(15, *N* = 956) = 72.49, *p* < 0.001, FDR‐adjusted *p* < 0.001, Cramér's *V* = 0.28, indicating a small‐to‐moderate effect size.

**TABLE 3:** Locations of reported workplace violence incidents.

Location of incident	Number of reports	Percentage
Patient room	*n* = 236	24.7%
Corridor	*n* = 285	29.8%
Seclusion room	*n* = 183	19.1%
Yard/outdoor area	*n* = 43	4.5%
Dining room	*n* = 16	1.7%
WC or washroom	*n* = 21	2.2%
Office (nurses' station/staff room)	*n* = 21	2.2%
Reception, procedure or examination room	*n* = 3	0.3%
Day room	*n* = 79	8.3%
Patient kitchen	*n* = 11	1.2%
Staff break room	*n* = 9	0.9%
Staff facilities/break room	*n* = 4	0.4%
Medication room	*n* = 3	0.3%
Recovery room	*n* = 4	0.4%
Other	*n* = 38	4.0%
Total	*n* = 956	100%

The distribution of violence severity varied notably depending on the location where the incident occurred. Severe incidents of workplace violence were most frequently reported in patient rooms (63.1%) and seclusion rooms (59.0%), followed by outdoor areas (60.5%) and day rooms (54.4%). Violence occurring in administrative spaces such as nursing stations (19.0%), break rooms (0.0%) and staff‐only areas was more likely to be classified as mild.

#### 4.3.2 Ward Type and Distribution of Incidents

The majority of workplace violence incidents were reported on the hospital's five most secure wards, known as assessment and stabilisation wards (75%). Five per cent of the incidents were reported on the three rehabilitation wards and on the five extended rehabilitation wards, 3% of the incidents were reported. On the adolescent ward, 18% of the workplace violence incidents were reported.

A chi‐square test found a significant association between hospital ward and workplace violence severity (mild vs. severe), *χ* ^2^(13, *N* = 956) = 199.27, *p* < 0.001, FDR‐adjusted *p* < 0.001, Cramér's *V* = 0.46, indicating a moderate‐to‐large effect size. This indicates that the distribution of mild and severe cases differed significantly across wards. For example, 78.2% of incidents reported on ward A were categorised as severe, compared to only 11.1% on ward B. Similarly, ward C also showed high rates of severe cases (71.5%), while wards D and E had considerably lower proportions (31.9% and 33.3%). Differences across wards may reflect case mix and security level. Severe incidents of workplace violence were particularly frequent on the women's ward and the ward for adolescent patients.

#### 4.3.3 Incidents Involving Outdoor Activities (Qualitative Analysis)

Qualitative incident narratives from incidents involving outdoor activities were analysed to identify the timing of incidents in relation to outdoor activities, the precipitating trigger, the form of violence and the incident outcome (Table 4). Consistent with the quantitative finding reported in Section 4.2.2, which shows a significant association between incidents involving outdoor activities and greater incident severity, we explore the mechanisms qualitatively here. Using inductive content analysis, four precipitating‐factor categories were identified in relation to workplace violence incidents: ‘denying/limiting patient needs/wants’ (*n* = 38/72), ‘interaction with staff members’ (*n* = 24/72), ‘interaction with other patients’ (*n* = 7/72) and ‘no specific reason’ (*n* = 18/72). Because more than one trigger could be identified in a single report, trigger categories were not mutually exclusive. Incidents involving outdoor activities were defined as reports in which the narrative linked the incident to outdoor activities before, during or after outdoor time; therefore, this subgroup was broader than incidents recorded with ‘yard/outdoor area’ as the physical location.

**TABLE 4:** Cross‐tabulation of incident classifications, trigger factors, forms of violence and outcomes for incidents involving outdoor activities, with exemplary quotes from HaiPro reports.

Timing of incidents involving outdoor activities	*n* (%)
Before outdoor activities	14/72 (19.4%)
During outdoor activities	36/72 (50.0%)
After outdoor activities	22/72 (30.6%)

Trigger of the workplace violence incident	*n* (%)	Exemplar quotes
Denying/limiting patient needs/wants	38/72 (52.8%)	‘After undressing, the patient followed the nurses to the seclusion room door and began demanding to be let out, using harsh language and an arrogant tone.’
Interaction with staff members	24/72 (33.3%)	‘The patient is offered supportive conversation in the room and also receives the necessary medication. After a brief discussion, however, the patient grabs a chair from their room and throws it against the wall.’
Interaction with other patients	7/72 (9.7%)	‘Other patients also asked the patient to stop, but the patient did not comply.’
No specific reason	18/72 (25.0%)	‘Quite suddenly, the patient lunged at the nurse who had opened the cabinet, with fists clenched and arms outstretched.’

Types of workplace violence	*n* (%)
Scratching/biting/spitting/bruising/hitting/kicking	52/72 (72.2%)
Insulting/name‐calling/verbal threats/yelling	11/72 (15.3%)
Pushing/shoving/waving away	7/72 (9.7%)
Breaking/throwing/slamming objects, items or equipment	1/72 (1%)

Outcome of the workplace violence incident	*n* (%)
Room seclusion	46/72 (63.9%)
Mechanical restraint (limb restraints)	6/72 (8.3%)
No seclusion	10/72 (13.9%)
Outcome unknown	10/72 (13.9%)

Based on the quantification of the qualitative data, 72.2% of incidents involving outdoor activities were classified in the ‘scratching/biting/spitting/bruising/hitting/kicking’ category, compared with only 35.9% across all hospital workplace violence reports. Taken together, the quantitative and qualitative analyses indicate that incidents involving outdoor activities were more often classified as severe and frequently involved physical violence, suggesting that outdoor activities may constitute a high‐risk routine context in this setting.

Based on the qualitative analysis, 50% of workplace violence incidents began during outdoor time, 31% after and 19% before. According to the qualitative narratives, incidents ended in room seclusion in 64% of cases, in mechanical restraint in 7%, without seclusion in 14% and in 14% the outcome was unclear. However, in some reports, it was not possible to determine with certainty whether the patient had already been in seclusion prior to the outdoor period.

### 4.4 Summary of Hypotheses

The findings provided support for H1–H4. After Benjamini–Hochberg FDR correction, the associations between workplace violence severity and time of day, incidents involving outdoor activities, incident location and ward remained statistically significant. In addition, the association between workplace violence severity and incident outcome (near miss vs. harm) remained statistically significant. The other chi‐square tests remained nonsignificant after correction. However, the associations for time of day and incidents involving outdoor activities were small and should be interpreted cautiously. The proportion of severe reports was highest during afternoon and daytime hours, incidents involving outdoor activities were more often classified as severe, severity differed by location and the proportion of severe incidents varied significantly across wards. These findings should be interpreted as associations rather than causal effects, particularly given the small effect sizes for time of day and incidents involving outdoor activities.

## 5 Discussion

This study examined the daily routines and activities, work and environmental factors associated with high‐risk workplace violence incidents affecting psychiatric nursing staff in a forensic psychiatric hospital. From a situational perspective, the findings suggest that severe workplace violence is not explained by patient‐related risk factors alone, but by the convergence of patient vulnerability, routine care activities, close staff–patient contact and environmental conditions that may limit immediate support or containment. The associations with daytime and afternoon periods, incidents involving outdoor activities and specific ward locations can therefore be understood as recurring high‐risk situations rather than isolated risk factors.

The hypotheses were broadly supported and the main associations remained statistically significant after FDR correction, although the strength of the associations varied. The strongest associations were observed for ward and incident location, whereas the associations for time of day and incidents involving outdoor activities were small. These findings suggest that severe workplace violence is best understood as arising from a combination of patient‐related, routine‐related and environmental factors rather than from any single risk factor.

Overall, 52% of the reports were categorised as severe workplace violence incidents, which is higher than the proportions reported by Kelly et al. (2015) and Renwick et al. (2019), although differences in definitions and reporting systems should be considered when comparing these figures. This finding highlights the considerable occurrence of severe workplace violence in forensic psychiatric care settings.

In this study, a statistically significant association was observed between severe workplace violence incidents and time of day: severe incidents occurred most frequently in the afternoon (57.6%) and during daytime hours (54.7%), whereas fewer such incidents were reported in the evening and at night. This may be explained by the fact that, on forensic psychiatric wards, most daily activities, such as meals, outdoor time, medication distribution and appointments with specialised staff (e.g., psychiatrists, psychologists), are scheduled for the morning and daytime, increasing patient–staff contact and potential triggers. Previous research in forensic psychiatric settings has shown that workplace violence incidents occur most often in the evening (Bader et al. 2014; Ridenour et al. 2015). Studies by Kuosmanen et al. (2022) and Iversen et al. (2016) further identified two peaks in violent incidents: one in the afternoon and another in the evening. This supports the interpretation that severe workplace violence may be more likely during active ward hours than at night, when patients are generally asleep, as reported in the studies by Kuosmanen et al. (2022) and Daffern et al. (2003). No statistical significance was found between day of the week and severity of workplace violence. From a routine activity perspective, time of day may therefore represent a proxy for exposure to staff–patient interaction, limit‐setting and ward transitions rather than an independent causal factor.

In this study, incidents involving outdoor activities were significantly associated with severe workplace violence. The qualitative incident narratives further showed that severe physical violence, such as hitting, kicking and biting, was reported markedly more often in incidents involving outdoor activities (72.2%) than across all hospital violence reports (35.9%). The narratives suggested that these incidents were often triggered by boundary‐setting, such as denying or limiting patient needs, as well as by staff–patient and patient–patient interactions. In the study hospital, outdoor activities are typically supervised by two staff members, which may limit rapid assistance and containment if a situation escalates. Thus, incidents involving outdoor activities may represent a specific routine context in which close staff–patient proximity, increased arousal and limited immediate containment coincide. These findings are consistent with previous research suggesting that workplace violence in forensic psychiatric settings often occurs during busy ward routines, such as mealtimes, medication rounds and staff shift changes (Bader et al. 2014) and that daily ward routines may be more strongly related to aggression than time of day alone (Bader et al. 2014; Kuosmanen et al. 2022). The findings also align with studies linking limited staffing resources and working alone to increased workplace violence risk (Edward et al. 2014; Mele et al. 2022; Weltens et al. 2021). Based on these findings, outdoor activities for forensic psychiatric patients should be assessed case‐by‐case, taking into account the patient's current mental state, recent behaviour and available staffing resources. However, because the observed statistical effect was small, incidents involving outdoor activities should be interpreted as one situational risk context rather than as an independent causal factor.

However, according to research, outdoor time for psychiatric patients is associated with well‐being, rehabilitation and recovery (Ross et al. 2022; McIntosh et al. 2022; Farrell et al. 2024). Therefore, outdoor access for forensic psychiatric patients should be restricted only if necessitated by the patient's mental condition or by compelling reasons related to hospital order or safety. This principle is also affirmed by the Council of Europe's CPT standards (Council of Europe 2015), which state that patients in psychiatric hospitals should have access to outdoor exercise.

In this study, workplace violence severity was statistically associated with incident location. Severe incidents frequently occurred in patient rooms, seclusion areas, outdoor spaces and common day rooms. This finding is consistent with previous research in forensic psychiatric settings, showing that workplace violence most often takes place in shared ward areas and patient rooms (Bader et al. 2014; Kuosmanen et al. 2022). Bader et al. (2014) note that violence in common ward areas may be reactive, whereas incidents in patient rooms may be premeditated. The higher frequency of violence in shared spaces can be explained by noise, increased interaction (Kuosmanen et al. 2022) and patient conflicts (Gudde et al. 2015; Bizzarri et al. 2020). Limited personal space and overcrowding further elevate the risk of workplace violence (Pulsford et al. 2013; Ireland et al. 2019; Weltens et al. 2021; Ross et al. 2022). While clear ward rules and structure can reduce the risk of incidents (Olsson et al. 2015), excessive boundary‐setting, such as smoking restrictions, may escalate conflicts and increase violence against staff (Ridenour et al. 2015; Bekelepi and Martin 2022). From an environmental psychology perspective, patient rooms, seclusion rooms and day rooms represent different environmental risk mechanisms. Patient rooms may involve close contact and reduced visibility, seclusion rooms often reflect already escalated situations and day rooms combine shared space, social density and patient–patient interaction.

Severe incidents were particularly frequent on the women's ward and the adolescent ward. This is consistent with previous studies linking younger age and female forensic patient groups with elevated aggression risk, although these patterns may reflect trauma histories, personality pathology, substance misuse, case mix and ward security level rather than gender or age alone (Kuivalainen et al. 2014; Verstegen et al. 2020; de Vogel et al. 2019; de Vogel and Didden 2022; Wolf et al. 2023). Similarly, Frowijn et al. (2024) found that staff‐targeted incident impact varied across forensic settings, supporting the interpretation that ward‐level differences reflect multiple patient, staff and organisational factors.

In the forensic psychiatric hospital under study, nurse managers process workplace violence reports, which are later reviewed with staff to clarify circumstances, contributing factors and prevention measures. Such post‐incident reviews support learning, restraint reduction and safety culture development (Hammervold et al. 2019; Kuosmanen et al. 2022).

Reporting behaviour is particularly relevant when interpreting temporal patterns. During busy daytime and afternoon periods, staff may have less time to report minor incidents, which could lead to a higher observed proportion of severe incidents in these periods. Previous research shows that workplace violence is often underreported, particularly when threats or violence are normalised as part of nursing work or when staff perceive that reporting does not lead to action (Archer et al. 2020; Babiarczyk et al. 2020; Mele et al. 2022; Spencer et al. 2023). Nevertheless, incident reporting is essential for improving workplace safety and developing preventive practices (Kuosmanen et al. 2022; Mele et al. 2022).

Taken together, these findings support a situational approach to violence prevention in forensic psychiatric nursing, in which preventive efforts are targeted not only at individual patient risk but also at recurrent high‐risk routines, time periods and ward locations.

## 6 Limitations

This study has several limitations. First, generalisability is limited because the data consists solely of HaiPro incident reports from a single forensic psychiatric hospital. Second, as the dataset only includes incidents reported by nursing staff, the findings may not extend to other professional groups, whose reporting thresholds and contexts can differ by role. Third, reporting incidents in HaiPro is voluntary and may vary by shift, ward culture and workload; for example, a heavy workload can contribute to underreporting and minor workplace violence is not always reported. Finally, multiple incidents may originate from the same patient, shift or ward, which may distort ward‐level counts of workplace violence reports.

The supplementary G*Power assessment indicated that the available sample was adequate for the main analyses of time of day, incident location and ward. However, although the association between incidents involving outdoor activities and severity remained statistically significant after FDR correction, the effect size was very small (*V* = 0.08) and detecting an effect of this size with 80% power would have required a larger sample than was available. Therefore, this finding should be interpreted cautiously and was supported with qualitative analysis.

In addition, reports initially classified as ‘Other situation, specify?’ were recoded as either mild or severe workplace violence by one researcher based on free‐text descriptions and the qualitative coding of incidents involving outdoor activities was also conducted by one researcher. Because formal interrater reliability was not assessed for these procedures, some risk of subjective classification or coding bias remains. As the analyses were based on routinely collected incident report data, it was not possible to control for patient‐level characteristics, staffing levels, exposure time, ward occupancy or repeated incidents involving the same patient. Therefore, the observed associations should not be interpreted as causal.

## 7 Conclusions

Workplace violence against psychiatric nurses is a recurrent problem in forensic psychiatric inpatient care. In line with previous research, severe reported incidents in this study were associated with specific routines, times and locations, particularly incidents involving outdoor activities, daytime and afternoon periods, patient rooms, seclusion rooms and day rooms. These findings support nurse‐led prevention strategies, including protocolised dynamic risk screening around outdoor time, location‐specific environmental controls and alignment of staffing and skill mix with high‐activity periods. Strengthening incident reporting, near‐miss review and violence‐prevention training may further support safer care environments and therapeutic continuity.

## Funding

The authors have nothing to report.

## Ethics Statement

Based on Finnish legislation (Finnish National Board on Research Integrity), ethical approval was not needed. The data for the study were obtained by applying for a research permit from the hospital in question. The hospital's chief physician granted the research permit and the data were provided to the researcher by the hospital's security manager. The hospital under study granted the researcher permission to conduct the research.

## Conflicts of Interest

The authors declare no conflicts of interest.

## Data Availability

Research data are not shared.

## References

[jpm70173-bib-0001] Abt, M. , P. Lequin , M. Bobo , T. Perrottet , J. Pasquier , and C. Bucher . 2022. “The Scope of Nursing Practice in a Psychiatric Unit: A Time and Motion Study.” Psychiatric and Mental Health Nursing 29, no. 2: 297–306. 10.1111/jpm.12790. PMC9290684 34310817

[jpm70173-bib-0002] Aerts, J. , M. Rijckmans , S. Bogaerts , and A. van Dam . 2023. “Establishing an Optimal Working Relationship With Patients With an Antisocial Personality Disorder. Aspects and Processes in the Therapeutic Alliance.” Psychology and Psychotherapy 96, no. 4: 999–1014. 10.1111/papt.12492. 37671752

[jpm70173-bib-0003] Archer, S. , B. Thibaut , L. Dewa , et al. 2020. “Barriers and Facilitators to Incident Reporting in Mental Healthcare Settings: A Qualitative Study.” Journal of Psychiatric and Mental Health Nursing 27, no. 3: 211–223. 10.1111/jpm.12570. 31639247

[jpm70173-bib-0004] Asikainen, J. , K. Vehviläinen‐Julkunen , E. Repo‐Tiihonen , and O. Louheranta . 2020. “Violence Factors and Debriefing in Psychiatric Inpatient Care: A Review.” Journal of Psychosocial Nursing and Mental Health Services 58, no. 5: 39–49. 10.3928/02793695-20200306-01. 32159814

[jpm70173-bib-0005] Awanic . 2020. Reporting System for Safety Incidents in Health Care Organizations. https://awanic.fi/haipro/eng/.

[jpm70173-bib-0006] Babiarczyk, B. , A. Turbiarz , M. Tomagová , R. Zeleníková , E. Önler , and C. D. Sancho . 2020. “Reporting of Workplace Violence Towards Nurses in 5 European Countries – A Cross‐Sectional Study.” International Journal of Occupational Medicine and Environmental Health 33, no. 3: 325–338. 10.13075/ijomeh.1896.01475. 32235948

[jpm70173-bib-0007] Bader, S. , and S. Evans . 2015. “Predictors of Severe and Repeated Aggression in a Maximum‐Security Forensic Psychiatric Hospital.” International Journal of Forensic Mental Health 14, no. 2: 110–119. 10.1080/14999013.2015.1045633.

[jpm70173-bib-0008] Bader, S. , S. Evans , and E. Welsh . 2014. “Aggression Among Psychiatric Inpatients: The Relationship Between Time, Place, Victims, and Severity Ratings.” Journal of the American Psychiatric Nurses Association 20, no. 3: 179–186. 10.1177/1078390314537377. 24904037

[jpm70173-bib-0009] Bekelepi, N. , and P. Martin . 2022. “Experience of Violence, Coping and Support for Nurses Working in Acute Psychiatric Wards.” South African Journal of Psychiatry 28: a1700. 10.4102/sajpsychiatry.v28i0.1700. PMC9210159 35747336

[jpm70173-bib-0010] Benjamini, Y. , and Y. Hochberg . 1995. “Controlling the False Discovery Rate: A Practical and Powerful Approach to Multiple Testing.” Journal of the Royal Statistical Society. Series B, Methodological 57, no. 1: 289–300. 10.1111/j.2517-6161.1995.tb02031.x.

[jpm70173-bib-0011] Bizzarri, J. V. , D. Piacentino , G. D. Kotzalidis , et al. 2020. “Aggression and Violence Toward Healthcare Workers in a Psychiatric Service in Italy: A Retrospective Questionnaire‐Based Survey.” Journal of Nervous and Mental Disease 208, no. 4: 299–305. 10.1097/NMD.0000000000001126. 32221184

[jpm70173-bib-0012] Bloemendaal, A. , A. Kamperman , A. Bonebakker , N. Kool , M. Olff , and C. Mulder . 2024. “Workplace Trauma and Professional Quality of Life in Clinical and Forensic Psychiatry: The CRITIC Study.” Frontiers in Psychiatry 15: 1228335. 10.3389/fpsyt.2024.1228335. 38495910 PMC10940400

[jpm70173-bib-0013] Bogaerts, S. , M. van Woerkom , Y. Erbaş , et al. 2021. “Associations Between Resilience, Psychological Well‐Being, Work‐Related Stress and Covid‐19 Fear in Forensic Healthcare Workers Using a Network Analysis.” Frontiers in Psychiatry 12: 678895. 10.3389/fpsyt.2021.678895. 34177662 PMC8226029

[jpm70173-bib-0014] Brenig, D. , P. Gade , and B. Voellm . 2023. “Is Mental Health Staff Training in de‐Escalation Techniques Effective in Reducing Violent Incidents in Forensic Psychiatric Settings? – A Systematic Review of the Literature.” BMC Psychiatry 23, no. 246: 246. 10.1186/s12888-023-04714-y. 37046228 PMC10099889

[jpm70173-bib-0015] Broderick, C. , A. Azizian , R. Kornbluh , and K. Warburton . 2015. “Prevalence of Physical Violence in a Forensic Psychiatric Hospital System During 2011–2013: Patient Assaults, Staff Assaults, and Repeatedly Violent Patients.” CNS Spectrums 20, no. 3: 319–330. 10.1017/S1092852915000188. 25937161

[jpm70173-bib-0016] Cohen, L. E. , and M. Felson . 1979. “Social Change and Crime Rate Trends: A Routine Activity Approach.” American Sociological Review 44, no. 4: 588–608.

[jpm70173-bib-0017] Council of Europe . 2015. CPT Standards: European Committee for the Prevention of Torture and Inhuman or Degrading Treatment or Punishment (CPT), CPT/Inf/E (2002) 1—Rev. 2015. Council of Europe. https://www.coe.int/en/web/cpt/standards.

[jpm70173-bib-0018] Cowman, S. , A. Björkdahl , E. Clarke , G. Gethin , and J. Maguire . 2017. “A Descriptive Survey Study of Violence Management and Priorities Among Psychiatric Staff in Mental Health Services, Across Seventeen European Countries.” BMC Health Services Research 59, no. 17: 59. 10.1186/s12913-017-1988-7. PMC5248457 28103871

[jpm70173-bib-0019] Daffern, M. , M. M. Mayer , and T. Martin . 2003. “A Preliminary Investigation Into Patterns of Aggression in an Australian Forensic Psychiatric Hospital.” Journal of Forensic Psychiatry & Psychology 14, no. 1: 67–84. 10.1080/1478994031000074306.

[jpm70173-bib-0020] de Vogel, V. , M. Bruggeman , and M. Lancel . 2019. “Gender‐Sensitive Violence Risk Assessment: Predictive Validity of Six Tools in Female Forensic Psychiatric Patients.” Criminal Justice and Behavior 46, no. 4: 528–549. 10.1177/0093854818824135.

[jpm70173-bib-0021] de Vogel, V. , and R. Didden . 2022. “Victimization History in Female Forensic Psychiatric Patients With Intellectual Disabilities: Results From a Dutch Multicenter Comparative Study.” Research in Developmental Disabilities 122: 104179. 10.1016/j.ridd.2022.104179. 35101719

[jpm70173-bib-0022] Edward, K. , K. Ousey , P. Warelow , and S. Lui . 2014. “Nursing and Aggression in the Workplace: A Systematic Review.” British Journal of Nursing 23, no. 12: 653–654. 10.12968/bjon.2014.23.12.653. 25039630

[jpm70173-bib-0023] Eisele, F. , E. Flammer , and T. Steinert . 2021. “Incidents of Aggression in German Psychiatric Hospitals: Is There an Increase?” PLoS One 16, no. 1: e0245090. 10.1371/journal.pone.0245090. 33400702 PMC7785246

[jpm70173-bib-0024] Elo, S. , M. Kääriäinen , O. Kanste , T. Pölkki , K. Utriainen , and H. Kyngäs . 2014. “Qualitative Content Analysis: A Focus on Trustworthiness.” SAGE Open 4, no. 1: 1–10. 10.1177/2158244014522633.

[jpm70173-bib-0025] Farrell, C. , K. Petersen , P. Hanzouli , and T. Nicholls . 2024. “Staff Supported Community Outings Among Forensic Mental Health Patients: Patient Characteristics, Rehabilitative Goals, and (The Absence of) Adverse Outcomes.” Frontiers in Psychiatry 15: 1382676. 10.3389/fpsyt.2024.1382676. 38628258 PMC11018879

[jpm70173-bib-0026] Finnish National Board on Research Integrity TENK . 2019. Ihmiseen Kohdistuvan Tutkimuksen Eettiset Periaatteet ja Ihmistieteiden Eettinen Ennakkoarviointi Suomessa. Tutkimuseettisen Neuvottelukunnan Ohje 2019. https://tenk.fi/sites/default/files/2021‐01/Ihmistieteiden_eettisen_ennakkoarvioinnin_ohje_2020.pdf.

[jpm70173-bib-0027] Fosse, R. , G. Eidhammer , L. Selmer , M. Knutzen , and S. Bjorkly . 2021. “Strong Associations Between Childhood Victimization and Community Violence in Male Forensic Mental Health Patients.” Frontiers in Psychiatry 1, no. 11: 628734. 10.3389/fpsyt.2020.628734. PMC7901946 33633598

[jpm70173-bib-0028] Frowijn, I. , E. Masthoff , J. K. Vermunt , and S. Bogaerts . 2024. “Transgressive Incidents Targeted on Staff in Forensic Psychiatric Healthcare: A Latent Class Analysis.” Frontiers in Psychiatry 15: 1394535. 10.3389/fpsyt.2024.1394535. 38832326 PMC11145633

[jpm70173-bib-0029] Glanz, A. , C. Sunnqvist , and K. Örmon . 2023. “The Time, Places, and Activities of Nurses in a Psychiatric Inpatient Context ‐ a Time and Motion Study With a Time‐Geographic Perspective.” Issues in Mental Health Nursing 44, no. 5: 387–396. 10.1080/01612840.2023.2194990. 37126738

[jpm70173-bib-0030] Gudde, C. B. , T. M. Olsø , R. Whittington , and S. Vatne . 2015. “Service Users' Experiences and Views of Aggressive Situations in Mental Health Care: A Systematic Review and Thematic Synthesis of Qualitative Studies.” Journal of Multidisciplinary Healthcare 8: 449–462. 10.2147/JMDH.S89486. 26491343 PMC4599636

[jpm70173-bib-0031] Hammervold, U. , R. Norvoll , R. Aas , and H. Sagvaag . 2019. “Post‐Incident Review After Restraint in Mental Health Care: A Potential for Knowledge Development, Recovery Promotion and Restraint Prevention. A Scoping Review.” BMC Health Services Research 19: 235. 10.1186/s12913-019-4060-y. 31014331 PMC6480590

[jpm70173-bib-0032] Hyvämäki, P. , S. Sneck , M. Meriläinen , M. Pikkarainen , M. Kääriäinen , and M. Jansson . 2023. “Interorganizational Health Information Exchange‐Related Patient Safety Incidents: A Descriptive Register‐Based Qualitative Study.” International Journal of Medical Informatics 174: 105045. 10.1016/j.ijmedinf.2023.105045. 36958225

[jpm70173-bib-0033] Ireland, C. , J. Ireland , N. Jones , S. Chu , and M. Lewis . 2019. “Predicting Security Incidents in High Secure Male Psychiatric Care.” International Journal of Law and Psychiatry 64: 40–52. 10.1016/j.ijlp.2019.01.004. 31122639

[jpm70173-bib-0034] Ireland, C. A. , S. Chu , J. L. Ireland , V. Hartley , R. Ozanne , and M. Lewis . 2021. “Extreme Stress Events in a Forensic Hospital Setting: Prevalence, Impact, and Protective Factors in Staff.” Issues in Mental Health Nursing 1−16: 418–433. 10.1080/01612840.2021.2003492. 34905419

[jpm70173-bib-0035] Iversen, C. , O. Aasen , I. Cüneyt Güzey , and A.‐S. Helvik . 2016. “Incidence of Violent Behavior Among Patients in Psychiatric Intensive Care Units.” European Journal of Psychiatry 30: 1.

[jpm70173-bib-0036] Jankovic, M. , J. J. Sijtsema , A. K. Reitz , E. D. Masthoff , and S. Bogaerts . 2021. “Workplace Violence, Post‐Traumatic Stress Disorder Symptoms, and Personality.” Personality and Individual Differences 168: 110410. 10.1016/j.paid.2020.110410.

[jpm70173-bib-0037] Johnston, I. , O. Price , P. McPherson , et al. 2022. “De‐Escalation of Conflict in Forensic Mental Health Inpatient Settings: A Theoretical Domains Framework‐Informed Qualitative Investigation of Staff and Patient Perspectives.” BMC Psychology 10, no. 30. 10.1186/s40359-022-00735-6. PMC8845398 35168682

[jpm70173-bib-0038] Kelly, E. , A. Subica , A. Fulginiti , J. Brekke , and R. Novaco . 2015. “A Cross‐Sectional Survey of Factors Related to Inpatient Assault of Staff in a Forensic Psychiatric Hospital.” Journal of Advanced Nursing 71, no. 5: 1110–1122. 10.1111/jan.12609. 25546118

[jpm70173-bib-0039] Kobayashi, Y. , M. Oe , T. Ishida , M. Matsuoka , H. Chiba , and N. Uchimura . 2020. “Workplace Violence and Its Effects on Burnout and Secondary Traumatic Stress Among Mental Healthcare Nurses in Japan.” International Journal of Environmental Research and Public Health 17, no. 8: 2747. 10.3390/ijerph17082747. 32316142 PMC7215457

[jpm70173-bib-0040] Kuivalainen, S. , K. Vehviläinen‐Julkunen , A. Putkonen , O. Louhenranta , and J. Tiihonen . 2014. “Violent Behaviour in a Forensic Psychiatric Hospital in Finland: An Analysis of Violence Incident Reports.” Journal of Psychiatric and Mental Health Nursing 21: 214–218. 10.1111/jpm.12074. 23634912

[jpm70173-bib-0041] Kuosmanen, A. , J. Tiihonen , E. Repo‐Tiihonen , M. Eronen , and H. Turunen . 2013. “Patient Safety Culture in Two Finnish State‐Run Forensic Psychiatric Hospitals.” Journal of Forensic Nursing 9: 207–216. 10.1097/JFN.0b013e318281068c. 24256983

[jpm70173-bib-0042] Kuosmanen, A. , J. Tiihonen , E. Repo‐Tiihonen , and H. Turunen . 2022. “Voluntary Patient Safety Incidents Reporting in Forensic Psychiatry—What Do the Reports Tell Us?” Journal of Psychiatric and Mental Health Nursing 29, no. 1: 36–47. 10.1111/jpm.12737. 33548085

[jpm70173-bib-0043] McIntosh, J. , B. Marques , and G. Jenkin . 2022. “The Role of Courtyards Within Acute Mental Health Wards: Designing With Recovery in Mind.” International Journal of Environmental Research and Public Health 19, no. 18: 11414. 10.3390/ijerph191811414. 36141687 PMC9517498

[jpm70173-bib-0044] Mele, F. , L. Buongiorno , D. Montalbo , et al. 2022. “Reporting Incidents in the Psychiatric Intensive Care Unit.” Journal of Nervous and Mental Disease 210, no. 8: 622–628. 10.1097/NMD.0000000000001504. 35394976 PMC10860884

[jpm70173-bib-0045] Mental Health Act (1116/1990) (Finland) . 1990. Finlex Data Bank, Ministry of Justice. https://www.finlex.fi/api/media/statute‐foreign‐language‐translation/689032/mainPdf/main.pdf?timestamp=1990‐12‐13T22%3A00%3A00.000Z.

[jpm70173-bib-0046] Moscovici, M. , F. Farrokhi , L. Vangala , A. Simpson , P. Kurdyak , and R. Jones . 2024. “Violence Risk Prediction in Mental Health Inpatient Settings Using the Dynamic Appraisal of Situational Aggression.” Frontiers in Psychiatry 15: 1460332. 10.3389/fpsyt.2024.1460332. 39720430 PMC11666551

[jpm70173-bib-0047] Newman, C. , M. Roche , and D. Elliott . 2021. “Exposure to Workplace Trauma for Forensic Mental Health Nurses: A Scoping Review.” International Journal of Nursing Studies 117: 103897. 10.1016/j.ijnurstu.2021.103897. 33647844

[jpm70173-bib-0048] Newman, C. , M. Roche , and D. Elliott . 2024. “Exposure to Patient Aggression and Health Outcomes for Forensic Mental Health Nurses: A Cross‐Sectional Survey.” Journal of Advanced Nursing 80, no. 3: 1201–1211. 10.1111/jan.15885. 37771198

[jpm70173-bib-0049] Niu, F.‐L. , S.‐F. Kuo , H.‐T. Tsai , C.‐C. Kao , V. Traynor , and K.‐R. Chou . 2019. “Prevalence of Workplace Violent Episodes Experienced by Nurses in Acute Psychiatric Settings.” PLoS One 14, no. 1: e0211183. 10.1371/journal.pone.0211183. 30677077 PMC6345477

[jpm70173-bib-0050] Ogonah, M. , A. Seydsalehi , D. Whiting , and S. Fazel . 2023. “Violence Risk Assessment Instruments in Forensic Psychiatric Populations: A Systematic Review and Meta‐Analysis.” Lancet Psychiatry 10, no. 10: 780–789. 10.1016/S2215-0366(23)00256-0. 37739584 PMC10914679

[jpm70173-bib-0051] Olsson, H. , Å. Audulv , S. Strand , and L. Kristiansen . 2015. “Reducing or Increasing Violence in Forensic Care: A Qualitative Study of Inpatient Experiences.” Archives of Psychiatric Nursing 29, no. 6: 393–400. 10.1016/j.apnu.2015.06.009. 26577553

[jpm70173-bib-0052] Pulsford, D. , A. Crumpton , A. Baker , T. Wilkins , and K. Wright . 2013. “Aggression in a High Secure Hospital: Staff and Patient Attitudes.” Journal of Psychiatric and Mental Health Nursing 20, no. 4: 296–304. 10.1111/j.1365-2850.2012.01908.x. 22486938

[jpm70173-bib-0056] Quality Network for Forensic Mental Health Services . 2021. Standards for forensic mental health services: Low and medium secure care (4th ed.). Royal College of Psychiatrists.

[jpm70173-bib-0053] Renwick, L. , M. Lavelle , K. James , D. Stewart , M. Richardson , and L. Bowers . 2019. “The Physical and Mental Health of Acute Psychiatric Ward Staff, and Its Relationship to Experience of Physical Violence.” International Journal of Mental Health Nursing 28, no. 1: 268–277. 10.1111/inm.12530. 30152005

[jpm70173-bib-0054] Ridenour, M. , M. Lanza , S. Hendricks , et al. 2015. “Incidence and Risk Factors of Workplace Violence on Psychiatric Staff.” Journal of Prevention, Assessment & Rehabilitation 51, no. 1: 19–28. 10.3233/WOR-141894. PMC4674794 24894691

[jpm70173-bib-0055] Ross, T. , J. Bulla , and M. Fontao . 2022. “Space and Well‐Being in High Security Environments.” Frontiers in Psychiatry 13: 894520. 10.3389/fpsyt.2022.894520. 35711591 PMC9195501

[jpm70173-bib-0057] Schaefer, L. 2021. “Routine Activity Theory.” In Oxford Research Encyclopedia of Criminology and Criminal Justice. Oxford University Press. 10.1093/acrefore/9780190264079.013.326.

[jpm70173-bib-0058] Seppänen, A. , P. Joelsson , A. Ahlgren‐Rimpiläinen , and E. Repo‐Tiihonen . 2020. “Forensic Psychiatry in Finland: An Overview of Past, Present and Future.” International Journal of Mental Health Systems 14, no. 29: 29. 10.1186/s13033-020-00362-x. 32322299 PMC7164302

[jpm70173-bib-0059] Spencer, C. , J. Sitarz , J. Fouse , and K. DeSanto . 2023. “Nurses' Rationale for Underreporting of Patient and Visitor Perpetrated Workplace Violence: A Systematic Review.” BMC Nursing 22, no. 134. 10.1186/s12912-023-01226-8. PMC10122798 37088834

[jpm70173-bib-0060] van Leeuwen, M. , and J. Harte . 2017. “Violence Against Mental Health Care Professionals: Prevalence, Nature and Consequences.” Journal of Forensic Psychiatry & Psychology 28, no. 5: 581–598. 10.1080/14789949.2015.1012533.

[jpm70173-bib-0061] Verstegen, N. , V. de Vogel , M. de Vries Robbé , and M. Helmerhorst . 2017. “Inpatient Violence in a Dutch Forensic Psychiatric Hospital.” Journal of Forensic Practice 19, no. 2: 102–114. 10.1108/JFP-04-2016-0020.

[jpm70173-bib-0062] Verstegen, N. , V. de Vogel , A. Huitema , R. Didden , and H. Nijman . 2020. “Physical Violence During Mandatory Psychiatric Treatment: Prevalence and Patient Characteristics.” Criminal Justice and Behavior 47: 771–789. 10.1177/0093854820924691.

[jpm70173-bib-0063] Weltens, I. , M. Bak , S. Verhagen , et al. 2021. “Aggression on the Psychiatric Ward: Prevalence and Risk Factors. A Systematic Review of the Literature.” PLoS One 16, no. 10: e0258346. 10.1371/journal.pone.0258346. 34624057 PMC8500453

[jpm70173-bib-0064] Wolf, V. , J. Mayer , I. Steiner , et al. 2023. “Risk Factors for Violence Among Female Forensic Inpatients With Schizophrenia.” Frontiers in Psychiatry 14: 1203824. 10.3389/fpsyt.2023.1203824. 37457783 PMC10347379

